# Advances in Mesenchymal Stem Cell-Derived Exosomes as Drug Delivery Vehicles

**DOI:** 10.3389/fbioe.2021.797359

**Published:** 2022-02-04

**Authors:** Dingyu Rao, Defa Huang, Chengpeng Sang, Tianyu Zhong, Zuxiong Zhang, Zhixian Tang

**Affiliations:** ^1^ The First Clinical College, Gannan Medical University, Ganzhou, China; ^2^ Department of Cardiothoracic Surgery, The First Affiliated Hospital of Ganna Medical University, Ganzhou, China; ^3^ Laboratory Medicine, First Affiliated Hospital of Gannan Medical University, Ganzhou, China

**Keywords:** exosomes, mesenchymal stem cells, drug delivery vehicle, tumor, nanocarrier (nanoparticle)

## Abstract

Exosomes are tiny vesicles with a double membrane structure that cells produce. They range in diameter from 40 to 150 nm and may contain a variety of biomolecules including proteins and nucleic acids. Exosomes have low toxicity, low immunogenicity, and the ability to encapsulate a wide variety of substances, making them attractive drug delivery vehicles. MSCs secrete large amounts of exosomes and hence serve as an excellent source of exosomes. MSCs-derived exosomes have regenerative and tissue repair functions comparable to MSCs and can circumvent the risks of immune rejection and infection associated with MSC transplantation, indicating that they may be a viable alternative to MSCs’ biological functions. In this review, we summarized the drug delivery methods and advantages of exosomes, as well as the advancement of MSC exosomes as drug carriers. The challenges and prospects of using exosomes as drug delivery vectors are presented.

## Introduction

Mesenchymal stem cells (MSCs) are a class of pluripotent stem cells capable of self-renewal and multidirectional differentiation. They are one of the most commonly employed stem cells. MSCs are derived from several tissues, including bone marrow, adipose tissue, muscle, and placenta (Jackson et al., 2013; Li et al., 2015; Frese et al., 2016). MSCs perform a number of biological functions, including tissue repair, immunosuppression, and neuroprotection (Yu et al., 2014). With low immunogenicity, multi-directional differentiation ability, in particular homing ability, it has significant research potential in cardiovascular diseases, nervous diseases, and hematopoietic diseases (Wang et al., 2018a). However, cells as drug carriers still face many problems such as uncertain differentiation accidents, cell embolism, infection, production, and storage (Su et al., 2021). Therefore, the drug delivery system based on MSCs has become one of the most attractive therapeutic methods. Many studies have suggested that the therapeutic effects of MSCs may be mediated by their paracrine secretion (Praveen Kumar et al., 2019; Kong et al., 2020). As the secretion of MSCs, the exosomes inherit the relative advantages of MSCs and overcome the problems of MSCs as drug carriers (Tang et al., 2021).

**FIGURE 1:**
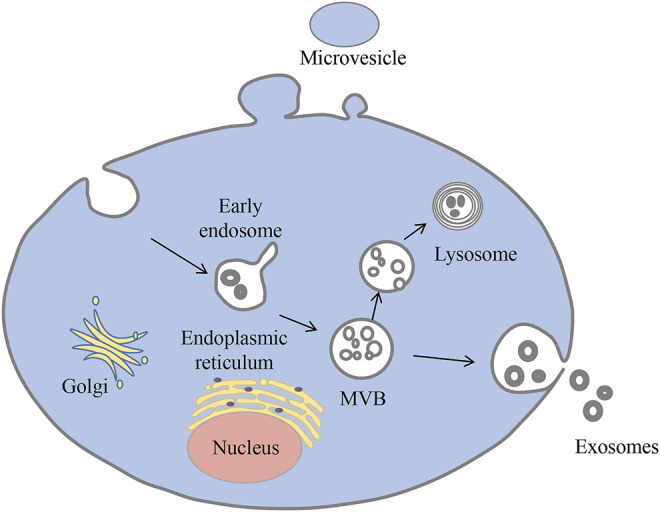
Biogenesis of exosomes. Exosomes begin as multivesicular vesicles (MVB), which invaginate the cytoplasmic membrane and then develop into early endosomes. The endosomes then bud inwards to form multivesicular bodies, which have two fates: one is to migrate to the cell surface and fuse with the plasma membrane, releasing the exosomes outside the cell in a cytosolic vomit; the other is to bind to lysosomes and degrade the contents.

Exosomes are nanoscale extracellular vesicles that organisms produce under normal physiological and pathological conditions. Exosomes were first discovered by Pan and Johnstone while investigating the maturation mechanisms of sheep reticulocytes into erythrocytes (Harding and Stahl, 1983; Pan and Johnstone, 1983; Pan et al., 1985). These vesicles originate from multivesicular bodies and are eventually released outside the cell (Latifkar et al., 2019) (Figure 1). Exosomes have been detected in a variety of body fluids, including blood, urine, breast milk, ascites, amniotic fluid, saliva, and cerebrospinal fluid (Wang et al., 2019). They can carry biologically active molecules such as nucleic acids and proteins and serve as messengers between cells, delivering their contents to the target cell (Hendrix et al., 2010; Gu et al., 2012; Weber et al., 2019).

MSCderived exosomes (MSC-Exos) were originally discovered by Lai et al. (2010) during the isolation of MSCs culture media. MSC-Exos have many unique characteristics, such as small size, low immunogenicity, long-circulating half-life, remarkable penetration, and biocompatibility. Furthermore, because of its particle size advantage, it can transport cargo molecules across biological barriers (such as the blood-brain barrier) (Chen et al., 2016a). It is one of the best choices for researchers to find drug carriers *in vivo* (Lai et al., 2013). Studies have shown that MSC-Exos possesses anti-fibrotic, anti-inflammatory, and pro-angiogenic effects with regenerative potential in myocardial tissue from ischemic injury in chronic myocardial infarction, acute and chronic renal injury, and fibrotic tissue (Gatti et al., 2011; Li et al., 2013; Yamaguchi et al., 2015). These features contribute to the potential of MSC-Exos as a natural drug delivery vehicle that can either accomplish the biological function of the package or achieve superior biological function than that of unmodified MSC-Exos.

## Isolation and Characterization of Exosomes

Currently, exosome isolation techniques include differential centrifugation, density gradient centrifugation, immunosorbent assay, precipitation, ultrafiltration, and size-exclusion chromatography (Cheruvanky et al., 2007; Lee et al., 2015; Lobb et al., 2015; Zeringer et al., 2015; Li et al., 2017a). Among them, differential centrifugation is the most frequently used method for exosome isolation and purification and is widely regarded as the gold standard for exosome isolation. Differential centrifugation and density gradient centrifugation make use of the density and size difference between exosomes and other components to separate exosomes using different centrifugal forces. Immunosorption occurs as a result of specific interaction between corresponding antibodies with exosomal membrane proteins. Precipitation chemicals, such as polyethylene glycol, are used to reduce the solubility of exosomes, causing them to precipitate. Ultrafiltration is a technique for separating exosomes based on their size differences using ultrafiltration membranes. Size exclusion chromatography employs polymer gel packings with varying particle sizes as the separation medium to separate exosomes based on their size properties. However, existing separation methods remain challenging. Microfluidic separation has recently been recognized as a method for rapidly and precisely isolating exosomes from small quantities of liquid samples (Liga et al., 2015).

At present, the most frequently used techniques for characterizing exosomes include electron microscopy, nanoparticle tracking analyzer, Western bolt (WB), and flow cytometry. Electron microscopy offers a high resolution and can determine the thickness, concentration, and particle size distribution of exosomes’ phospholipid bilayer (Nojima et al., 1993). Nanoparticle tracking analysis estimates exosome concentration, particle size distribution, and other information by tracking the trajectory of individual particles (Xu et al., 2011). WB is a traditional exosome characterization method that uses high-quality monoclonal antibodies to identify antigen-presenting proteins (e.g. CD63, CD81, CD9, TSG101, ALIX, etc.) on the surface of exosomes to improve the reliability of identification results (Tian et al., 2018a). Flow cytometry offers the advantages of high speed, high statistical accuracy, and high practicability, making it an advanced analytical tool for exosome heterogeneity analysis, exosome-based diagnostic markers research, and therapeutic agent development.

## Exosome Drug Delivery Mode

To date, exosome loading drug approaches are divided into two categories: extracellular and intracellular loading strategies.

### Extracellular Loading Strategy

**FIGURE 2:**
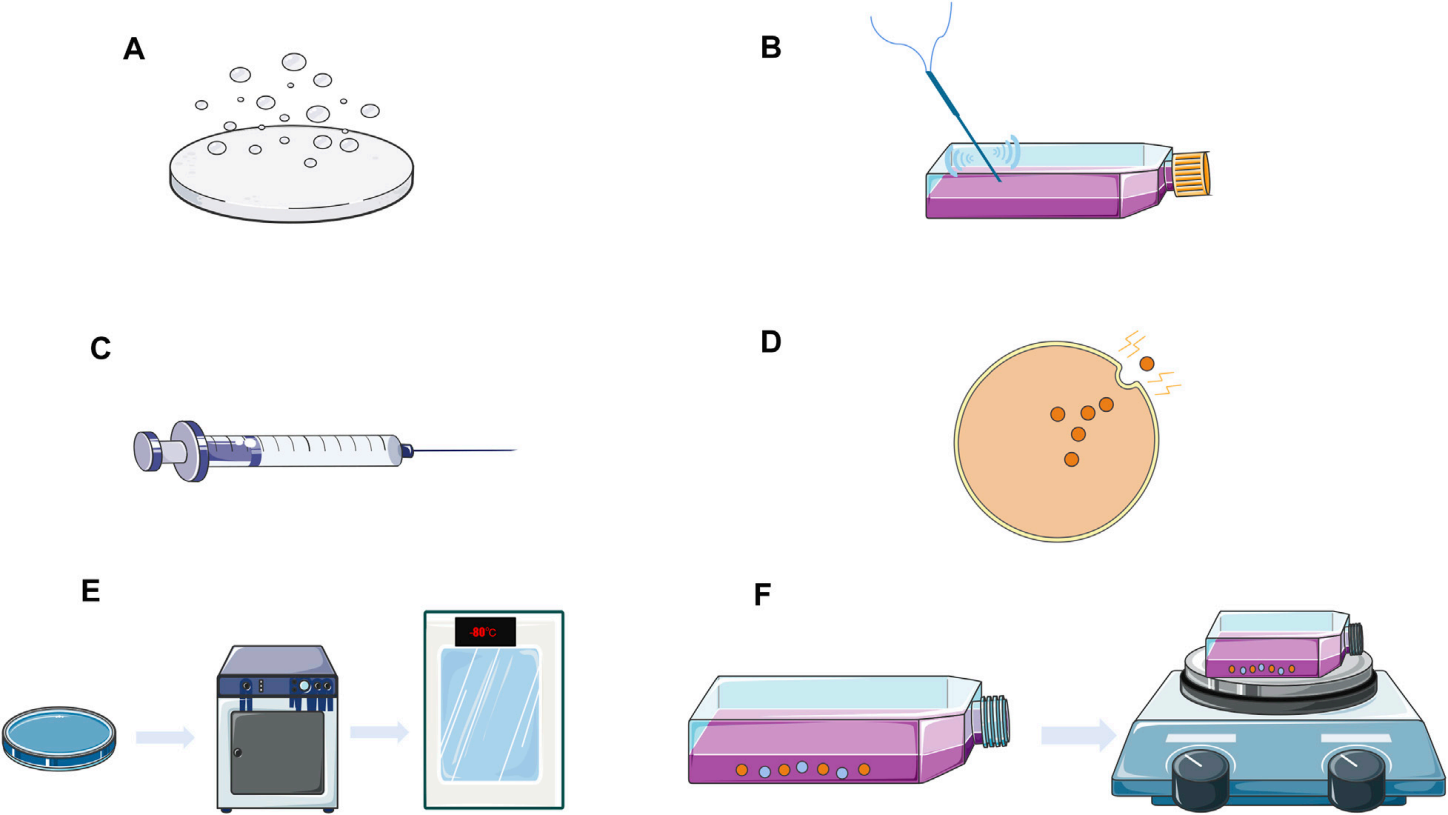
Exosomes extracellular drug delivery strategy. **(A)** Drug co-incubation; **(B)** Acoustic processing; **(C)** Squeeze method; **(D)** Electroporation; **(E)** Freeze-thaw; **(F)** Saponin-assisted loading

Extracellular drug loading entails the purification and isolation of exosomes from donor cells followed by the loading of the desired drug into the exosomes using several methods such as incubation, electroporation, sonication, extrusion, freeze-thaw, and saponin-assisted loading (Figure 2). The table below summarizes the advantages, disadvantages, and applications of various extracellular drug delivery methods (Table 1).

**TABLE 1:** Advantages, disadvantages, and applications of various extracellular drug delivery methods.

Method	Advantages	Disadvantages	Application	Reference
Drug co-incubation	Simple, no additional equipment required	Low efficiency	Curcumin, paclitaxel, peroxidase, siRNA, porphyrin	34
Electroporation	Loadable with large molecules	Disruption of exosome membrane integrity	siRNA, paclitaxel, porphyrin	35, 36
Acoustic treatment	Higher efficiency	The membrane is easily deformed and ineffective against hydrophobic substances	siRNA, paclitaxel, porphyrin, miRNA	38, 39
Extrusion method	Higher efficiency	Some damage to the exosome membrane Causes cytotoxicity	Porphyrins, peroxidase	40
Freeze-thaw method	Applicable to most substances	Exosome aggregation and low efficiency	Catalase, paclitaxel	41
Saponin	High drug loading capacity	The exocrine body produces pores, control, and cleaning	Peroxidase, porphyrin	42, 43

The simplest method of exosome loading is drug co-incubation, which involves simply combining the isolated exosome with the drug. Loading is driven by drug concentration, and the efficiency of loading by this method is dependent on the drug’s lipophilicity as well as the concentration gradient (Sun et al., 2010). The primary disadvantage of this method is its low loading efficiency.

The electroporation technique of drug loading disrupts the electric field of the phospholipid bilayer of the exosome, forming small pores in its membrane, and allowing the drug to pass into the vesicle. The integrity of the vesicle membrane is then restored, allowing the formation of drug-loaded vesicles. Studies have shown that electroporation has been effective in loading siRNAs into exosomes (Alvarez-Erviti et al., 2011). Wahlgren et al. (2012) showed that electroporation results in a higher siRNA loading than chemical transfection. However, electroporation has been challenged as a method of loading siRNA into exosomes, with Kooijmans et al. (2013) stating that electroporation destroys exosome integrity and is significantly less effective than co-incubation.

Acoustic treatment, in which exosomes from donor cells are combined with the drug and sonicated by a probe sonicator, results in the drug flowing into the exosome due to ultrasound-induced membrane deformation. The following are some examples of ultrasound treatments for drug loading: Kim et al. (2016) loaded paclitaxel into exosomes released from macrophages using ultrasound treatment. This method offers a high loading efficiency and sustained drug release while having no effect on exosome protein and lipid content. Lamichhane et al. (2016) also used ultrasound to deliver functional small RNAs into exosomes isolated from HEK293T and MCF-7 cells.

The extrusion method involves combining the exosomes with the drug, then loading the mixture into a syringe-based lipid extruder and extruding through a membrane with a pore size of 100–400 nm at a controlled temperature. During extrusion, the exosome membrane is disrupted and forcefully mixed with the drug, allowing the drug to be loaded into the exosome. Fuhrmann et al. loaded porphyrins into the exosomes by extrusion. However, it was demonstrated that the exosome extrusion method of drug delivery caused cytotoxicity, while other methods of preparing exosome carriers did not (Antimisiaris et al., 2018).

The freeze-thaw method entails incubating the drug with exosomes at room temperature, then freezing the mixture at −80°C or in liquid nitrogen, and then thawing it at room temperature. To load the drug into the exosomes, the procedure is consecutively repeated three times (Sato et al., 2016). This method allows peroxisomes, therapeutic RNA, and other components to be loaded into exosomes. However, this method may cause exosomes to aggregate, resulting in a significantly poorer loading efficiency than that ultrasound or extrusion methods.

Saponin is a surfactant molecule that co-incubates with exosomes to generate pores in the exosome membrane, enhancing exosome-membrane permeability. To generate peroxidase-loaded exosomes, Haney et al. (2015) mixed peroxidase with exosomes, added saponin and placed them on an oscillator for 20 min at room temperature. This method may also be used to load hydrophilic molecules into exosomes, such as porphyrins (Fuhrmann et al., 2015). This method offers a high loading efficiency and prevents protease degradation.

### Intracellular Drug Delivery Strategy

Intracellular drug loading is the co-culture of drug with donor cells or chemically transfected donor cells loaded into the donor cells, and when the drug enters the exosome and is released from the donor cells, the drug-loaded exosome is isolated and purified.

Although the loading efficiency of drug co-culture with donor cells is uncontrollable, it has been used in more research due to the ease with which the drug may be loaded. Pascucci et al. (2014) encapsulated paclitaxel (PTX) into mouse mesenchymal stromal cells, washed, and incubated them with fresh media. After 48 h, exosomes were recovered from PTX-loaded cells. Exosomes loaded with PTX had a strong inhibitory impact on CFPAC-1 human pancreatic cells as compared to exosomes obtained from cells in the untreated group.

Transfection is the most commonly used and effective method for loading therapeutic proteins or oligonucleotides into exosomes. Akao et al. (2010) successfully loaded therapeutic miRNAs into TH-1 macrophage secretory exosomes. Similarly, Ohno et al. (2013) demonstrated that exosomes loaded with miRNA could effectively target recipient cells when their surfaces were modified with specific peptides, as well as that intravenous injection of exosomes could reduce tumor growth at tumor accumulative sites.

## Advantages of Exosomes as Drug Carriers

A liposome is an ultramicroscopic spherical carrier formulation formed by a lipid bilayer. It possesses the ability to load lipophilic and hydrophilic drugs and to exhibit targeting effects by the attachment of targeting ligands to its surface (Zhou et al., 2013a). While the liposome is a relatively mature drug carrier, it has some limitations, including the toxicity of the synthetic liposome membrane and the low biocompatibility of targeted ligand (Sercombe et al., 2015).

Exosomes outperform synthetic liposomes as drug carriers. 1) Exosomes are actively secreted by living cells and can be considered as natural liposomes, overcoming the limitations of synthetic liposomes; 2) Exosomes are derived from the organism, making them less immunogenic and possessing good tolerability and safety (Chen et al., 2016b); 3) Exosomes can cross the blood-brain barrier and enter the brain circulation, allowing for non-invasive treatment of intracerebral diseases (Long et al., 2017); 4) Exosomes have intrinsic homing properties and can also be artificially modified to express specific molecules or to improve their targeting ability (Li et al., 2017b).

MSCExos has unique advantages as a drug carrier. Although primary cells derived from MSCs are commonly used in clinical practice can produce a large number of exosomes, they have limited proliferation capacity for large-scale production, and the exosomes produced may have batch-to-batch variation, necessitating repeated testing and validation in multiple batches and increasing production costs (Fang et al., 2019). Chen et al. (2011) found that using c-myc transfected human embryonic stem cell-derived MSC (hESC-MSC) may enhance the proliferation rate and reduce the time required to produce MSCs without compromising the exosome quality. Transfected hESC-MSC may produce exosomes in the milligram range, indicating the possibility of producing biologically beneficial exosomes. Subsequently, a previous study compared the ability of c-myc-transfected hESC-MSC, skeletal muscle cell lines, HEK cells, small airway epithelial cell lines, and THP1 cell lines to produce exosomes. By analyzing CD81 levels, this study found that hESC-MSC produced at least 10-fold the amount of CD81 ^+^ exosomes produced by other cell lines (Yeo et al., 2013).

In addition, exosomes can be functionally modified to improve their use as carriers. Exosome functionalization can increase exosome circulation duration, improve exosome intercellular transport efficiency, and promote better targeting of exosomes (Lu et al., 2018). At present, the commonly used targeted modification approach is to use genetic engineering to transfect the gene encoding the targeted peptide into exosome source cells, resulting in exosomes carrying the targeted peptide (Shamili et al., 2018). Furthermore, some studies have used a covalent approach to exosome modification, such as bioorthogonal copper-free azide-alkyne cycloaddition to attach functional ligands to exosomes, which has been shown to have no significant effect on exosome structural integrity and interaction with receptor cells and can be used for rapid and large-scale production of functionalized exosomes (Tian et al., 2018b). In addition to covalent approaches, non-covalent modifications such as electrostatic interactions, receptor-ligand binding, and hydrophobic reactions can be utilized to modify the surface of exosomes (Armstrong et al., 2017).

## Application of MSC-Exos as a Drug Delivery Vehicle

MSCExos has similar physiological functions to MSCs, indicating its potential for application as a therapeutic agent. Additionally, MSC-Exos can act as a drug delivery carrier, which is increasingly used in the treatment of cardiovascular diseases, neurological diseases, and malignant tumors.

### Diseases of the Cardiovascular System

At present, an increasing number of researchers are interested in the effect of MSC-Exos on cardiovascular disease. MSC-EXOS has been shown to reduce myocardial ischemia-reperfusion injury (MI/R) through multiple signaling pathways, such as activation of the Wnt/β-catenin signaling pathway (Cui et al., 2017). The AMPK/mTOR and Akt/mTOR pathways also induce autophagy in cardiomyocytes (Liu et al., 2017). Furthermore, MSC-Exos therapy increased the ATP and NADH levels in MI/R hearts, reduced the degree of oxidative stress, and significantly reduced local and systemic inflammatory responses (Arslan et al., 2013). Cardiac stem cells (CSCs) pretreated with MSC-Exos have a better survival rate and promote long-term recovery of cardiac function in rat models of myocardial infarction (Zhang et al., 2016a).

MiR-132 can modulate endothelial cells during angiogenesis, however, its safe delivery *in vivo* remains an unresolved issue. Ma et al. (2018) investigated whether MSC-Exos could be used to treat myocardial ischemia through miR-132 delivery. Exosomes loaded with miR-132 were able to significantly increase the lumen-like structure of endothelial cells *in vitro*, while exosome-pretreated human umbilical vein endothelial cells exhibited increased angiogenic potential *in vivo*. In addition, implantation of Mir-132-coated exosomes into the ischemic heart of mice, significantly increased new angiogenesis in the periinfarct region and protected cardiac function. Following a myocardial infarction, the therapeutic impact of transplanted MSCs may be enhanced by exosomes loaded with Mir-125b-5p61, which improves autophagy (Xiao et al., 2018). Furthermore, the recombinant adenovirus-mediated Mir-486 gene was used to modify MSCs, and it was discovered that the mir-486 level in MSC-Exos was increased, and MSC-Exos with high expression of Mir-486 was found to promote cardiomyocytes proliferation and migration while inhibiting cardiomyocytes apoptosis. It is anticipated that it will be a novel strategy for cardiac regeneration and repair (Fang et al., 2018).

Interference with the environment in which exosomes are produced (e.g., hypoxia) has been discovered to affect the components secreted by exosomes. Zhu et al. (2018a) investigated whether hypoxia-treated MSCs-derived exosomes (ExoH) were superior to those produced under normoxic conditions (ExoN) in terms of myocardial repair. ExoH-treated mice showed greater survival rates, smaller scars, and improved cardiac function in myocardial infarction experiments in mice. Further research found that ExoH exhibited greater levels of miR-210 expression than ExoN, as well as expression of neutral sphingomyelinase 2, which is essential for exosome secretion. Similarly, Zhu et al. (2018b) discovered that miR125b-5p delivered by MSC-Exos under hypoxic conditions promote ischemic heart repair by improving cardiomyocyte apoptosis. In addition, they developed a novel drug delivery vector by collocating ExoH with an ischemia myocardial targeting peptide, increasing the specificity of drug delivery in ischemic diseases. It was also shown that ExoH significantly reduced apoptosis and reactive oxygen species generation in CSCs following oxidative stress injury compared to ExoN, most likely due to its higher miR-214 expression, although the overall mechanism of action remains unknown (Wang et al., 2018b).

### Neurological Diseases

The blood-brain barrier prevents the flow of endogenous molecules, exogenous biological agents, and immune-monitoring cells such as macrophages, therefore preserving central system homeostasis (Zhou et al., 2018). When neurological lesions occur, therapeutic drugs cannot reach the corresponding target cells due to the protective effect of the blood-brain barrier, limiting the treatment of neurological diseases. MSC-Exos increases functional recovery, neurosynaptic remodelling, neurogenesis, and angiogenesis in a rat stroke model, and represents a novel treatment option for stroke (Xin et al., 2013). Small porcine adipose-derived MSCs and their associated exosomes reduce the size of the cerebral infarct zone and enhance neurological function in rats, in an acute ischemic stroke model, with a significant safety profile (Chen et al., 2016b). In addition, MSC-Exos loaded with peroxidase was found to successfully cross the blood-brain barrier and ameliorate the disease state of Parkinson’s disease (Haney et al., 2021). MSC-Exos was also found to protect retinal pigment epithelial cells from blue light stimulation and improve laser-induced retinal damage by down-regulating vascular endothelial growth factor-A (He et al., 2018).

Loss of Retinal Ganglion Cells (RGC) and their axons is a major cause of blindness. Mead and Tomarev (2017) demonstrated for the first time that MSC-Exos was effective in protecting RGC. MSC-Exos promoted RGC survival and axon regeneration in the rat optic nerve crush model while preventing RGC axon loss and dysfunction to some extent. To further examine the mechanism of RGC protection by MSC-Exos, this study transfected MSCs with siRNA to silence the Argonaute-2 gene (a key miRNA effector) and isolated the exosomes generated. It was discovered that the exosome successfully delivered its “cargo” to the inner retina and that the effect was miRNA-dependent, with the therapeutic effect of MSC-Exos being reduced when Argonaute-2 was knocked out.

### Malignant Tumors

MSCExos has also garnered considerable attention for its potential use in the treatment of malignant tumors. Breast cancer is currently treated mostly by surgical excision, chemotherapy, radiotherapy, and hormone therapy (Yu et al., 2015). MSC-Exos can address the drawbacks and hence has significant potential as a targeted delivery vehicle for breast cancer treatment. Lee et al. (2013) demonstrated that MSC-Exos significantly down-regulated the expression of vascular endothelial growth factor in breast cancer cells, thereby inhibiting angiogenesis both *in vitro* and *in vivo*. Lin et al. (2013), on the other hand, suggested that MSC-Exos could promote breast cancer cell migration via the Wnt signaling pathway. Therefore, the mechanism by which MSC-Exos acts on breast cancer must be further explored to maximize MSC-Exos’s therapeutic effects.

Dormant breast cancer cells induce MSCs to release exosomes carrying various miRNAs (e.g., miR-222/223), therefore promoting quiescence and conferring drug resistance in a proportion of cancer cells (Bliss et al., 2016). MSC-Exos was found to suppress breast cancer tumorigenesis *in vitro* and *in vivo* through the delivery of miRNA-142-3p inhibitors. MSC-Exos successfully delivered miRNA-142-3p inhibitors *in vitro,* reduced miRNA-142-3p and miR-150 levels, and increased transcription of regulatory target genes APC and P2X7R. *In vivo*, MSC-Exos can deliver inhibitory oligonucleotides to tumor tissue, thus down-regulating miRNA-142-3p and miR-150 expression levels (Naseri et al., 2018). In addition, miR-100 delivered by MSC-Exos was able to inhibit angiogenesis *in vitro* by regulating the mTOR/HIF-1α/VEGF signaling axis in breast cancer cells, thereby influencing the behavior of breast cancer cells (Pakravan et al., 2017).

TNF-Related Apoptosis Induced Ligand (TRAIL) is one of the potential antitumor drugs for preclinical research due to its ability to induce selective apoptosis in a variety of tumor cells without causing toxic effects on normal cells. Shamili et al. (2018) utilized non-viral vectors to introduce plasmids encoding TRAIL -GFP into MSCs. The findings indicate that MSCs-derived TRAIL-loaded exosomes (Exo-TRAIL) inhibit melanoma progression by promoting massive necrosis of cancer cells and that their anti-tumor effect is dose-dependent. Pascucci et al. (2014) loaded MSC-Exos with paclitaxel by co-incubating high doses of paclitaxel with MSCs, which inhibited the proliferation of pancreatic cancer cells. Kalimuthu et al. (2018) found that paclitaxel-loaded MSC-Exos mimics were effective in inhibiting the growth of breast cancer *in vivo* than unencapsulated MSC-Exos mimics, and because the mimics were easily isolated, they provided a novel drug delivery vehicle for the treatment of breast cancer.

In addition, MSC-Exos can be utilized to treat different types of tumors by delivering endogenous or exogenous miRNAs, proteins, etc., Table 2 summarizes the various cargoes delivered by MSC-Exos for different tumor treatments (Table 2).

**TABLE 2:** A summary of the various cargoes delivered by MSC-Exos for different oncology treatments.

Cancer type	Delivery drug type	Reference
Breast cancer	miRNA-142-3p, miR-150, Paclitaxel	78, 79, 80
Melanoma	TRAIL	56
Pancreatic cancer	Paclitaxel	44
Ovarian cancer	hsa-miR-124-3p	86
Glioblastoma	anti-miR-9, miR-124	88, 89
Lung cancer	PDGFD, siGRP78	90, 91

However, exosomes may also negatively affect chemotherapy treatment by shuttling chemotherapeutic drugs away from the target cancer cells (Kalluri, 2016). Study confirmed the presence of cisplatin and doxorubicin in post-treatment cancer cell-derived exosomes (Yin et al., 2012). In addition, HER-2^+^ exosomes produced by HER-2 overexpressing breast cancer cells inhibited trastuzumab-induced anti-proliferative activity (Ciravolo et al., 2012). Thus, removing HER-2^+^ exosomes from the circulation of HER-2 overexpressing breast cancer patients has a positive effect on trastuzumab therapy (Marleau et al., 2012). Depletion of exosomes from the blood of cancer patients may also improve exosome-mediated immune tolerance (Ichim et al., 2008). MSC-Exos may provide therapeutic benefits for cancer patients, but more research is needed to understand the combined effects of MSC-Exos on the body. As a result, there are no Food and Drug Administration (FDA)-approved exosome products available, and this is also the case in China.

### Others

Studies have shown that MSC-Exos can repair osteochondral damage and can better restore steroid-induced early ischemia necrosis of the femoral head by delivering mutant HIF-1α (Zhang et al., 2016b; Jiang et al., 2018). In a model of acute liver injury induced by carbon tetrachloride, the antioxidant and hepatoprotective effects of MSC-Exos were superior to those of the commonly used hepatoprotective agent bifenthix (Chen et al., 2018). In a mouse model of autoimmune hepatitis, MSC-Exos encapsulated with miR-223 significantly reduced serum levels of ALT, AST, and pro-inflammatory cytokines, as well as mRNA levels of these cytokines in the liver compared to MSC-Exos (Jia et al., 2018). Furthermore, in a model of cisplatin-induced acute kidney injury, MSC-Exos was able to prevent nephrotoxic injury caused by cisplatin by delivering 14-3-3ζ, and it had the effect of repairing kidney injury both *in vivo* and *in vitro* (Zhou et al., 2013b; Jeppesen et al., 2019).

## Discussion

Exosomes as an emerging drug delivery vehicle have become a research hotspot in recent years. It exhibits unique benefits due to its biological properties such as non-toxicity and non-immunogenicity. This study focused on the isolation and characterization of exosomes, outlined the drug delivery modalities and advantages of exosomes, and summarized the application of MSC-Exos as a drug carrier in cardiovascular system diseases, neurological diseases, and malignancies.

Extracellular vesicles of the same size or larger than exosomes have been reported to develop in many cell types via the outgrowth of plasma membranes or membrane extensions (e.g., microvilli, filamentous pseudopods, cilia, and flagella) from the main body of the cell. Extracellular vesicles of equivalent size to exosomes have the same biophysical properties as exosomes in terms of size, density, and membrane localization, and hence existing methods cannot effectively distinguish them.

A significant advancement in exosome research in recent years has been the growing recognition that extracellular vesicles, such as exosomes, include many distinct granule subtypes, each of which may have intriguing functions in intercellular communication. Despite growing interest in this field, understanding of the cellular and molecular mechanisms that control extracellular vesicle biogenesis, release, uptake, and function is limited. The technical difficulties in isolating and characterizing specific granule subtypes is a significant limitation in accurately describing extracellular vesicles since the methods currently used result in systematic co-isolation of extracellular vesicles of different subcellular origins. Therefore, while numerous articles use the term “exosomes” to refer to preparations of extracellular vesicles isolated from larger extracellular vesicles by physical processes, they are more likely to refer to a mixture of tiny extracellular vesicles that are exosomal and non-exosomal in nature. as a result, unless their multivesicular origin is identified, the generic term “small extracellular vesicles” may be preferred.

Jeppesen et al. (2019) recently reported on the use of high-resolution density gradient separation and direct immunoaffinity capture techniques to precisely characterize RNA, DNA, and protein fractions in exosomes, and other non-vesicular material. Extracellular RNA, RNA-binding proteins, and other cellular proteins were found to be differentially expressed in exosomes and non-vesicular compartments, but no Argonaute 1, Argonaute 2, Argonaute 3, Argonaute 4, glycolytic enzymes, or cytoskeletal proteins were detected in exosomes. Membrane-linked protein A1 (annexin A1) was discovered to be a unique marker for microvesicles shed directly from the plasma membrane. They also discovered that tiny extracellular vesicles do not function as carriers of DNA release. Instead, they proposed a novel model for active extracellular DNA secretion that relies on autophagy and the multivesicular endosome rather than exosomes. As a result, these findings imply that the composition of exosomes should be re-evaluated, providing a framework for a better understanding of the heterogeneity of extracellular vesicles.

Current understanding of the physiology, diversity, internalization, and transport of molecular cargoes in extracellular vesicles, including exosomes, is still very limited, therefore it impossible to draw precise conclusions about the mechanisms by which extracellular vesicles interact with and modify recipient cells. To achieve advances in the field of extracellular vesicles, investigations must be conducted in an integrated way, encompassing molecular, cellular, and functional characterization, so that various extracellular vesicle subtypes in a particular experimental system may be compared to the greatest extent possible. These approaches are critical for determining which molecules or mechanisms are exclusive to various extracellular vesicle subtypes and which are relevant to all extracellular vesicle subtypes.

Therefore, despite the great potential of exosomes as drug carriers, there are still significant challenges: 1) Exosome composition and mode of action of exosomes must be well understood to ensure their safety and efficacy, and formulation composition and mode of action must be validated. 2) The need to improve and refine isolation and purification procedures, as well as increase the efficiency of exosome production. 3) The choice of which cell-derived exosomes to use as drug carriers must be targeted to reduce off-targeting, side effects, and clearance. If these challenges are adequately addressed, exosomes will be the next generation of drug delivery systems. Exosomal drug carriers are expected to be widely employed in the clinic in the near future.
